# Virtual Superheroes: Using Superpowers in Virtual Reality to Encourage Prosocial Behavior

**DOI:** 10.1371/journal.pone.0055003

**Published:** 2013-01-30

**Authors:** Robin S. Rosenberg, Shawnee L. Baughman, Jeremy N. Bailenson

**Affiliations:** 1 Private Practice, Stanford, California, United States of America; 2 Department of Communication, Stanford University, Palo Alto, California, United States of America; Hungarian Academy of Sciences, Hungary

## Abstract

**Background:**

Recent studies have shown that playing prosocial video games leads to greater subsequent prosocial behavior in the real world. However, immersive virtual reality allows people to occupy avatars that are different from them in a perceptually realistic manner. We examine how occupying an avatar with the superhero ability to fly increases helping behavior.

**Principal Findings:**

Using a two-by-two design, participants were either given the power of flight (their arm movements were tracked to control their flight akin to Superman’s flying ability) or rode as a passenger in a helicopter, and were assigned one of two tasks, either to help find a missing diabetic child in need of insulin or to tour a virtual city. Participants in the “super-flight” conditions helped the experimenter pick up spilled pens after their virtual experience significantly more than those who were virtual passengers in a helicopter.

**Conclusion:**

The results indicate that having the “superpower” of flight leads to greater helping behavior in the real world, regardless of how participants used that power. A possible mechanism for this result is that having the power of flight primed concepts and prototypes associated with superheroes (e.g., Superman). This research illustrates the potential of using experiences in virtual reality technology to increase prosocial behavior in the physical world.

## Introduction

Experiences in virtual reality (VR) can be powerful–the user can feel as if he or she were actually “present” in the VR world. For instance, people walking on a log across a virtual chasm may know intellectually that they are in a VR world, but nonetheless experience many of the psychological symptoms they would experience if asked to cross an actual chasm (e.g., stress as measured by skin conductance [1]). Similarly, people with a fear of flying who therapeutically experience a virtual plane flight are helped to overcome their fears as much as people who therapeutically experience a real flight as part of a fear-of-flying course or therapy (see Rizzo & Kim [2] for a thorough discussion of therapy with virtual reality). The effects of virtual experiences can endure; for example: the plane-phobic person is able to take plane flights months later. [3] In this paper, we discuss how giving participants an enhanced ability in VR–the power to fly using their arms–affected helping behavior after they were out of the VR world.

Recent work has examined how people come to “inhabit” or embody their *avatars*, which are virtual representations of themselves [4]. For example, Slater and colleagues [5] demonstrated that male participants experienced a so-called “body transfer illusion” even when their avatars were female. Moreover, a subsequent study by Kilteni, Normand, Sanchez-Vives, and Slater [6] demonstrated that participants could successfully transfer themselves into avatars that are shaped fundamentally differently from them, for example ones with arms much longer than human physical arms. These findings are particularly relevant to the current study in which participants occupy avatars that can perform similarly nonhuman feats–flying like Superman.

We examined whether inhabiting an avatar that is helpful would cause someone to become more altruistic. Studies show that computer games that induce the user to behave in prosocial ways can lead users to engage in helping behaviors in the real world after the game is over. For example, Gentile and colleagues [7] helped to pioneer research on this topic with a series of studies with Singaporean children, Japanese children and adolescents, and U.S. undergraduate students. In the first study, Singaporean children reported which games they played most often and how often players hurt or helped others in the game. The children then completed a series of scales assessing their aptitude for prosocial behavior. In this study, the researchers found that prosocial game exposure was positively related to prosocial behavior and traits. In their second study, Japanese children’s video game habits and prosocial behaviors were tested over 3 to 4 months to assess the notion that habitually playing prosocial video games would increase prosocial behavior. Again, participants were surveyed on how often they played games with prosocial content (i.e., scenes in which characters help troubled persons). Results for the second study demonstrated a correlation between prosocial behavior and prosocial gaming. Finally, in their third study, college students participated in an experiment designed to assess causality of that relationship. Participants were randomly assigned to play either a prosocial video game or a neutral video game for 20 minutes. Next, participants were told to assign a partner 11 tangram puzzles out of 30 (10 easy, 10 medium, and 10 hard.) They were told that if their partner could complete 10 puzzles out of 11 then the partner would win a $10 gift certificate. Thus, partners could help each other by assigning easy puzzles or hurt each other by assigning hard ones. The researchers found a significant effect of game type on behavior. Those who played prosocial games were more likely to help their partner than those who played neutral games.

In another study [8], participants were randomly assigned to play one of four video games: Lemmings, City Crisis, Tetris, and Lamers. Lemmings and City Crisis were deemed prosocial, the first being a game in which the participant must guide groups of small beings and save them by leading them to an exit, and the second being a game where the player acts as a helicopter pilot who has to rescue citizens from burning houses, support the police, and perform other helpful behaviors. Tetris was deemed a neutral game, and Lamers was classified by the experimenters as an aggressive form of Lemmings in which all beings must be killed before reaching the exit. After game play, participants who played a prosocial game were more likely to help pick up pencils that were “accidentally” spilled than participants who played the neutral or aggressive game. In a subsequent study [9], the researchers explored how prosocial video games increased the accessibility of prosocial thoughts. By using prosocial word recognition and recording response time they found that playing prosocial video games primed prosocial thoughts into semantic memory more so than when playing neutral video games.

Prosocial computer or console games can enhance empathy [10], and immersive virtual reality appears to be at least equally suited to understanding and promoting empathy [11]. Virtual reality allows users to psychologically “become” the avatars, due to the realistic tracking of movements and perceptual similarity of avatar to self [12]. For example, Hershfield and colleagues [13] described studies in which virtual doppelgangers (virtual humans bearing a strong resemblance to the self) were created and manipulated to test how seeing an older version of oneself would affect the amount of money saved for retirement. In a series of studies testing this relationship, the researchers found that when participants interacted via their aged self, they were more likely to accept later monetary rewards over immediate ones. By implementing a series of controls, the researchers concluded that showing participants an older version of themselves opened up cognitive channels that allowed participants to be more concerned for their future and thus save more for retirement.

Similarly, Fox and Bailenson [14] found that viewing a virtual representation of one’s self exercising or not exercising, and subsequently watching the virtual representation lose and gain weight because of it, increased the likelihood that one will exercise more in real life. Specifically, when exposing participants to a representation of their own avatar running versus their own avatar loitering, researchers found that participants in the running condition exhibited higher levels of exercise in the 24-hour period following the study than those who viewed their avatar loitering. This fosters the idea that virtual reality can influence behaviors for some time after treatment.

In this vein, the current study sought to discover whether simply experiencing a virtual enhanced ability (i.e., the power of flight), and the unstated but implicit concepts that go along with such an enhanced ability (e.g., *super*power) would lead participants subsequently to be more helpful. Specifically, we wanted to explore whether implicit but powerful priming of the concept “superhero” would lead to subsequent helping behavior. Other research has suggested this manipulation may be successful. For example, Nelson and Norton [15] found that participants who were primed with the category “superhero” were more likely to volunteer subsequently than participants in the control group. Participants were primed to think about either superheroes as a category, or about a prototype of that category–the superhero Superman. Participants were then asked how many hours they would be willing to volunteer at an organization. Participants primed with the general category of superheroes were significantly more likely to volunteer their time compared to participants in the other conditions. The current research takes these ideas one step further by allowing participants to *embody* a superhero ability rather than just think about the concept, and in measuring the helping effect afterward, we have the added strength of a behavioral measure rather than a self-reported measure.

We specifically examined two variables in VR and their effect on subsequent helping behavior out of the VR world: (1) whether simply experiencing in VR an enhanced virtual ability (flying) would lead people to engage in prosocial behavior after the VR experience, and (2) whether performing a helping task in a virtual environment would lead people to help on an unrelated task after being in the virtual environment.

## Materials and Methods

### Ethics Statement

The experiment was approved by the Stanford University Institutional Review Board, and all participants gave their written informed consent. The study was performed according to institutional ethics and national standards for the protection of human participants.

### Materials

**Figure 1:**
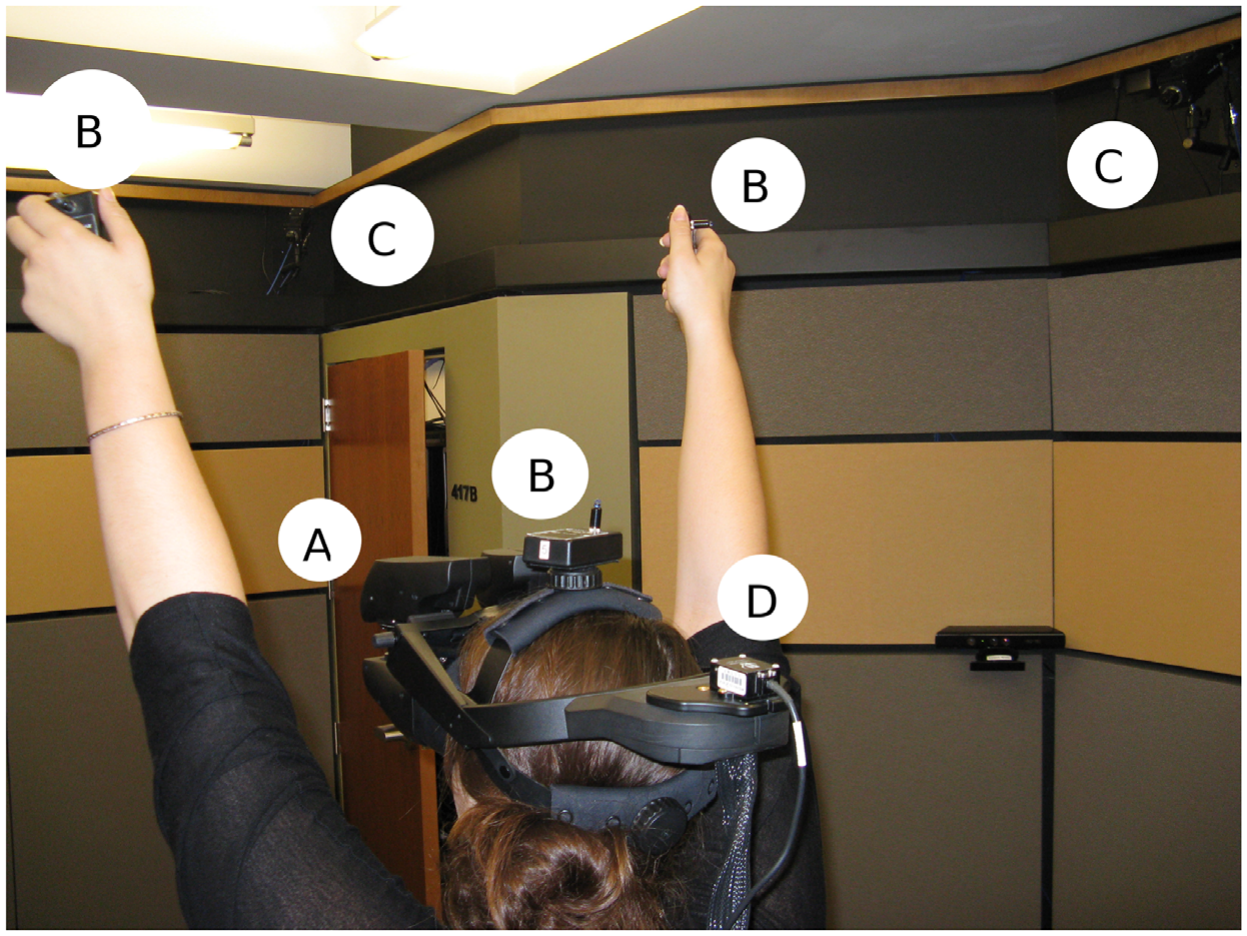
Participant flying in VR. This figure shows: (A) The labels the Head Mounted Display, which renders the virtual world on two screens, one for each of the participant’s eyes; (B) The optical tracking markers are labeled. One marker is placed on the participant’s head and two are placed in the participant’s hands. These markers track X,Y, Z position; When the participant raises her hands above her head, she flies higher in the virtual city; (C) One of the eight motion-capture cameras that track the optical marks; and (D) The orientation tracker for head rotation.

The immersive virtual environment was created with a Python OpenGL software toolkit called Vizard (Worldviz), which was rendered in real-time by a high-performance Dell Precision T7500 running Windows 7 with 12GB of memory and a NVIDIA GTX 680 graphics card with 1.5GB of video memory. Participants in the immersive virtual environment (IVE) viewed the world through an nVisor SX111 head-mounted display (HMD), a fully immersive virtual reality helmet that allows for three-dimensional stereoscopic views of a rendered environment at a resolution of 2056×1024 pixels and a field-of-view of 111 degrees diagonal. An orientation sensor (Intersense3 Cube accelerometer) mounted to the HMD operating at 180 hertz with a 4 millisecond latency rate was used to track participants’ physical head movements (pitch, roll, and yaw) and update their rendered first-person perspective viewpoint in the IVE. Additionally, three points on participants’ physical body positions–the head and each hand–were tracked (X, Y and Z) using an optical infrared camera system (Worldviz PPT-H) operating at 180 hertz with a 20 millisecond latency rate and a precision of 0.25 millimeters (see Figure 1). To add additional sensory modalities, virtual sound was also added to the IVE by utilizing a three-dimensional ambisonic sound auralizer (Worldviz), which combines 24 channels of audio information (22 speakers and 2 subwoofers) that communicates with the IVE. This allows sounds to move in the physical space and match objects from where they are moving in the IVE. Additionally, low-frequency bass sounds that are played utilize an additional 16-subwoofer (ButtKickers) setup that provides tactile haptic vibration to participants by literally shaking the floor underneath them.

### Experimental Design

Participants were assigned to receive either the virtual power of flight, akin to Superman’s ability to fly (the super flight condition), or to fly as a passenger in a helicopter (the helicopter flight condition). Participants were also assigned either to a helping condition to find a young, lost diabetic child in need of life-saving insulin immediately, or a touring condition to navigate and explore the virtual city. Thus, the study was a two-by-two design with assignment to one of four possible groups (see Table 1): Superpowered helper; helicopter helper; superpowered tourist; helicopter tourist.

**Table 1:** Number of Participants Per Condition.

	Super Flight	Helicopter Flight
**Helping**	N = 16	N = 17
**Touring**	N = 14	N = 13

A total of 74 people participated in the study. Nine participants were lost due to technical problems with the tracking algorithm or the video recording system. Two participants ended the study early due to motion sickness. Three participants specifically mentioned the helping dependent variable when asked what the purpose of the study was, and were removed due to possible demand characteristics. Hence the final sample included 30 females and 30 males. Table 1 shows the final distribution across conditions. Note that the unequal number of participants across cells occurred because of various technical problems or issues with participants (such as motion sickness during the VR portion of the study, or correctly guessing the hypothesis of the study).

**Figure 2:**
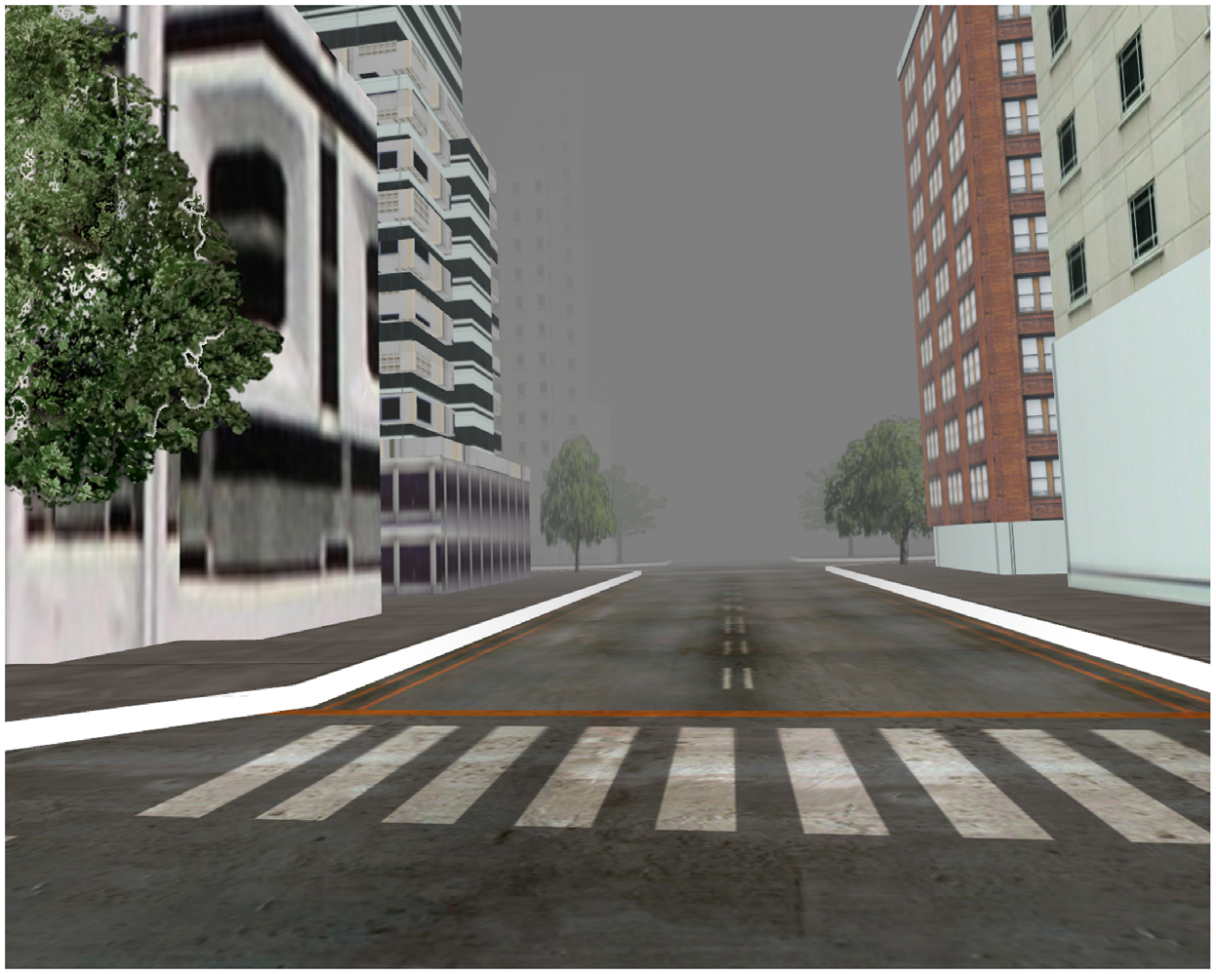
Foggy virtual city.

The virtual city was designed to be foggy to prevent participants from flying too high above the buildings to see the ground. The city repeated with a recursive algorithm that wrapped their position back to the beginning when they exited the city area to allow for flight that covered distances larger than the three-dimensional model of the city, which was approximately a ten-by-ten-block square. The virtual city was intentionally devoid of cars or people (aside from one child in the helping conditions), and all participants were told that an earthquake necessitated the evacuation of a city in order to explain the lack of people. Figure 2 shows a screenshot of the virtual city.

The study was conducted in three stages. Stage 1 was an immersive virtual experience in which the participants were either given the ability to fly or “positioned” in a virtual helicopter. Stage 2 was the collection of the behavioral dependent variable, measured after the VR experience: The experimenter knocked over a cup of pens, ostensibly by accident, in order to allow the participant an opportunity to help by picking them up. During Stage 3, participants went to a small testing room and completed several surveys.

### Procedures

Participants were assigned to a condition based on gender and sequential placement among the four conditions. The first male and female participants were each assigned to superpowered helping. The next three male and female participants were assigned to each of the three remaining conditions. For example, after the first female participant completed the superpowered helping condition successfully, the next female would be assigned to the helicopter helping condition, the third female would complete the superpowered touring condition, the fourth female would complete the helicopter touring condition, and then the sequence would begin again. The same method was used for male participants. Participant dropout due to technical failures caused the sequence to begin again at the first condition. Each participant spent approximately 20 minutes completing the study. Participants were compensated with partial credit for an experimental participation requirement for class. After signing a consent form, participants were lead to a room in which they were immersed in virtual reality (Stage 1).

Participants in the super flight conditions were given the following flight instructions (for the scripts for each condition, see Text A in File S1): “In this virtual environment, you will have the ability to fly. I will explain how flight works. Sensors will be placed in each of your hands and they will allow you to control the direction and speed of your flight. To take off from the ground, you will lift your hands all the way above your head, and to land you can drop your hands to your sides. In general, where your hands are pointed is the direction in which you will fly. If you’d like to turn, you may move your hands and your entire body, like this [experimenter demonstrates.] To control your speed, you must control the distance between your hands. The closer your hands are together, the faster you’ll fly and the further your hands are apart, the slower you’ll fly. Your task will be explained to you once you are immersed in virtual reality.” The flight algorithm tracked the three infrared lights: one on the head, and one on each wrist of the participant. A directional vector was calculated between the head light and a point in the direct center between a participant’s two wrists. Participants were then translated in the virtual world along the directional vector at a speed determined by the distance between the two wrist lights; farther distance created a slower speed of translation whereas closer distance creates a faster speed.

In the two helicopter conditions, participants were merely told that they were to be a passenger in a helicopter and their task would be explained once immersed in virtual reality. Their field of view varied only as a function of their head movements (i.e., they did not control translation of the helicopter but could look around the vehicle and out the window).

After putting on the HMD each participant heard a recorded set of instructions played through the speakers in the room. For the two helping conditions, the instructions were similar. Specifically, in the superpowered helper condition, participants heard the following instructions: “There has been an earthquake warning and the city has been evacuated. A child has been unaccounted for and the parents have informed authorities that their child is diabetic and will go into shock without insulin. You have a vial of insulin in your pocket. Your task is to fly through the city to find the child and deliver the insulin, saving the child’s life. As soon as you see the child, call out. You must indicate to the experimenter that you’ve found the child so please clearly announce that you see the child when the child comes into view. You may now begin your search. Lift your arms above your head to take off from the ground.” With the exception of a few words and the instruction to lift his or her arms, the participants in the helicopter helper condition received the same instructions.

For the touring conditions, the instructions were different. In the superpowered touring condition, participants heard, “In this environment, you will use your ability to fly to explore a virtual city. You may begin by lifting your arms above your head to take off from the ground.” In the helicopter touring condition, participants heard, “In this environment, you will be a passenger in a helicopter as it explores the virtual city. Your helicopter tour will begin now.” Participants in the helicopter conditions were told that a helicopter pilot was seated behind a partition next to them (the pilot was intentionally not visible, controlling for the possible confound of social presence) and that the pilot was navigating the helicopter through the city.

The timing and, in part, the flight path of the participants were controlled across conditions using a yoking technique. The orientation and position of the flight path of any participant in the superpowered helping condition was recorded, and used as input for the exact flight path and vehicle orientation for a subsequent participant in the corresponding helicopter condition. In the helicopter conditions, the flight paths were yoked directly to the flight paths taken by participants in the superpowered helping condition. However, participants in the superpowered touring condition controlled their own flight path so copying tracking data was not possible. Instead, the exact flight *duration* was set based on that of the corresponding participant in the superpowered helping condition. If the participant in the latter condition took exactly three minutes and fourteen seconds to find the child, then the participant in superpowered touring condition would have exactly three minutes and fourteen seconds to tour the city. In sum, the duration of each helicopter condition were directly yoked to that of the participants in corresponding flying conditions, and the duration of each superpower touring condition was yoked to the duration of a previous superpower helping condition.

**Figure 3:**
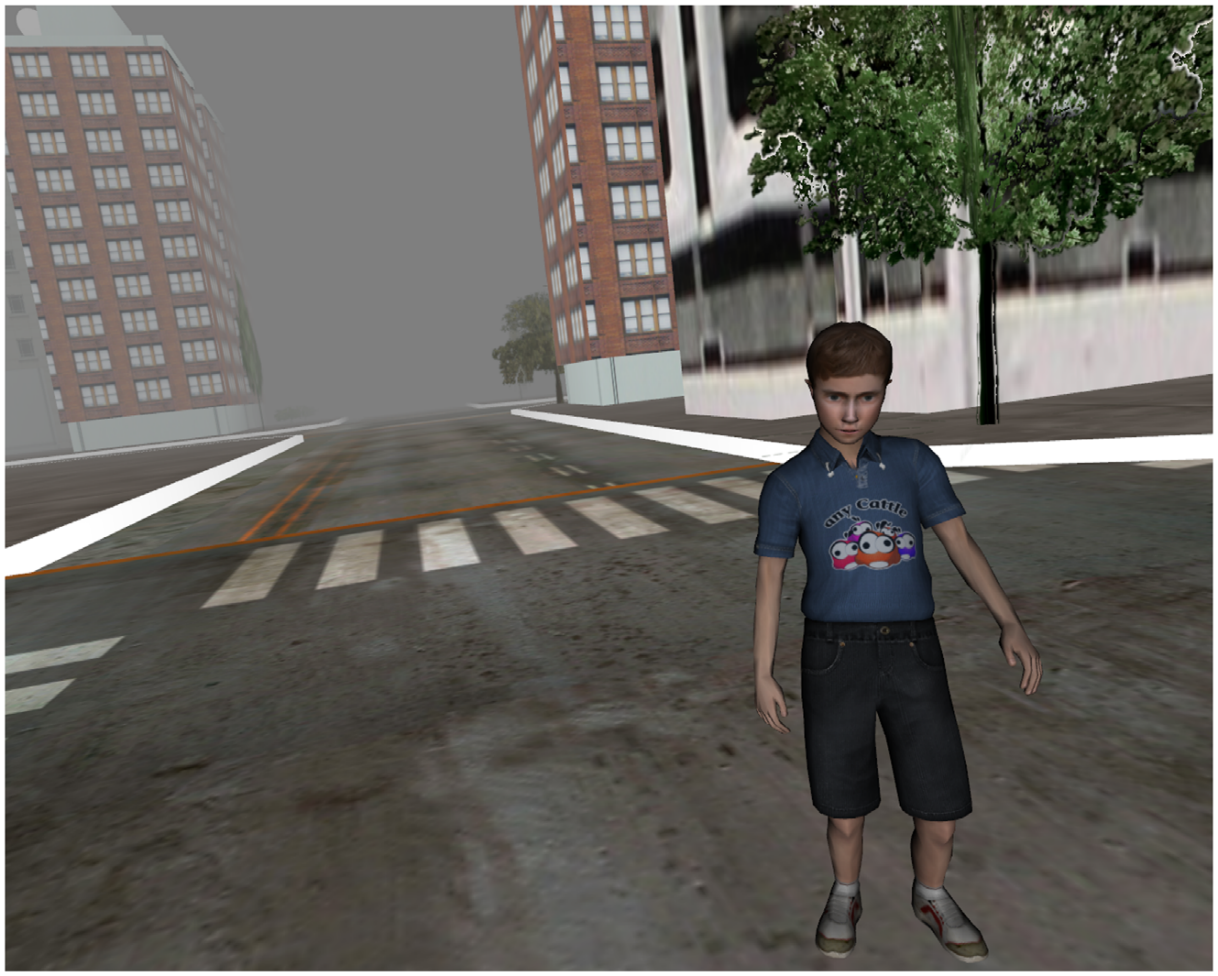
Virtual Child After Being Found and Saved.

In both helping conditions, participants were given three minutes to fly around the city looking for the child. After three minutes, the child would appear at the intersection nearest the participant. Participants had been instructed to call out when they saw the child, after which an end key was pressed in the control room and participants lost control over their flight and were guided by the software over to the child where an end sequence played, explaining that the participant had saved the child’s life; the child’s gender was never specified, and the virtual image of the child was intentionally designed to appear androgynous. (See Figure 3 for an illustration of the virtual child in the city.).

**Figure 4:**
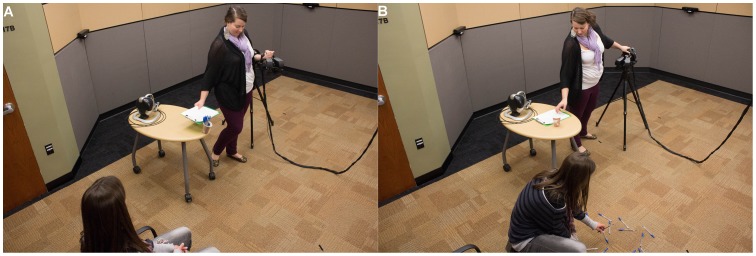
The Experimenter Knocks Over The Pens And A Participant Kneels To Help Pick Up Pens. In Panel A, the experimenter is “accidentally” knocking over the pens; in Panel B, a participant gets out the chair and kneels to help pick up the pens. Note: The images are slightly blurry as the surveillance cameras do not capture in high resolution.

After completing the virtual task, the participant was taken out of the HMD and asked to have a seat in a chair nearby while the experimenter put away the virtual reality equipment. While the experimenter fumbled with the equipment, she “accidentally” knocked over a cup of 15 pens sitting on a table approximately four feet in front of the participant’s chair (Stage 2). The trained experimenter then waited five seconds before attempting to pick up the pens, giving the participant time to help. If the participant did not get up to help within those five seconds, the experimenter picked up the pens one at a time, still giving the participant the opportunity to help. See Figure 4A and 4B for photographs of the pens procedure. After the pen task was complete, the experimenter took the participant into a separate room to complete several questionnaires (Stage 3).

### Behavioral Variables

#### Time to help

This variable was adapted from Greitemeyer and Osswald [8]. All participants were videotaped and three independent coders were instructed to note the exact point in the video that participants stood up from their chairs to help. The average correlation among the three coders was.95. The average time to get up was 4.34 seconds (*SD* = 6.03).

#### Number of pens picked up

This was also adapted from Greitemeyer and Osswald [8]. The three coders counted how many of the pens participants picked up before the experimenter finished picking up the pens after a standard five second delay from when the pens were spilled. The average correlation among the three coders was.95. The average number of pens picked up (out of 15) was 12.54 pens (*SD* = 3.88).

### Self-Report Variables

#### Simulator Sickness Questionnaire (SSQ)

The SSQ [16] contains 16-items to measure the physical aftereffects of being in the virtual environment. This measure was included to ensure that helicopter riding participants were not sicker than flying participants due to the passive nature of their locomotion. Scale reliability was sufficient, Cronbach’s Alpha = .86.

#### Environmental Presence

A five-item scale to measure environmental presence, loosely adapted from Bailenson, Swinth, Hoyt, Persky, Dimoy, and Blascovich [17]; for the exact wording of the questions, see Text B in File S1. Presence refers to the sense that the virtual environment, and the person’s interactions in that environment, feel “real.” In general, it measures a sense of engagement with the environment. Presence served as a tool to examine the mechanism behind possible differences in helping effects. Scale reliability was sufficient, Cronbach’s Alpha = .86.

#### Intention to Help

A 17-item questionnaire adapted for a college population from a self-report subscale about helping behavior of the Prosocial Orientation Questionnaire [18] (POQ), used with young children in a study on prosocial video game [7]. The original survey contained statements such as “I would help my family if they were in need” and “I always do things to make my teachers happy.” We re-phrased some questions to fit our college sample and eliminated ones that were irrelevant for this age group. For the exact wording of the questions, see Text C in File S1. Scale reliability was low but acceptable, Cronbach’s Alpha = .71.

#### Purpose of the Experiment

Finally, participants were asked to write about their thoughts about the experiment’s hypothesis. Based on previous work using behavioral measures subject to demand characteristics, we wanted to ensure that people did not explicitly associate the pen accident with the experiment. Three participants explicitly mentioned the pen task in their written response and were subsequently removed from the sample before data analysis.

### Predictions

Based on the results of previous studies on prosocial computer games and prosocial behavior, we had two hypotheses: (1) a main effect for motion type such that flying participants would be more helpful than helicopter participants, (2) a main effect for task, such that participants who helped the child would be more helpful than those who toured. In addition, we wanted to examine the possibility of an interaction such that participants in the superpowered helping condition would be the most helpful. We also predicted a larger effect from behavioral measures of helping than from the self-report measures of helping, as previous research has demonstrated similar findings [19], [20].

## Results

### Time to Help

Table 2 shows the means and standard deviations for the number of pens picked up across condition. There were six participants who chose not to help with the pens at all; all were in one of the two helicopter conditions). For these participants, we coded their delay as the number of seconds it took for the experimenter to pick up all the pens. We performed an ANOVA with task type and motion type as independent variables. There was a significant effect of motion type, *F*(1,56) = 7.50, *p*<.01, *Partial Eta Squared* = .12, such that flying participants helped more quickly than helicopter participants. (Because we substituted the maximum time in for participants who did not pick up any pens, and because all of the participants who did not pick any up were in the helicopter conditions, the variance was larger in the helicopter condition than the flying condition. It should be noted that the Levene’s statistic for this test was significant, indicating unequal variances. The ANOVA, however, is a robust test, especially when cells are all relatively equal in sample size.) Neither the main effect of task type, *F*(1,56) = .03, *p*<.86, *Partial Eta Squared* = .00, nor the interaction, *F*(1,56) = .05, *p*<.82, *Partial Eta Squared* = .001, were significant.

**Table 2:** Means And Standard Deviations For Time To Help.

	Super flight	Helicopter flight
**Helping**	2.26 (.91)	6.17 (8.01)
**Touring**	2.19 (.73)	6.81 (8.97)

Standard deviations are in parentheses.

### Number of Pens

Table 3 contains the mean and standard deviation of number of pens picked up across condition. We ran an ANOVA with task type and motion type as independent variables. There was a significant effect of motion type, *F*(1, 56) = 4.74, *p*<.03, *Partial Eta Squared* = .08, such that flying participants picked up more pens than helicopter participants. Neither the main effect of task type, *F*(1,56) = .99, *p*<.32, *Partial Eta Squared* = .02, nor the interaction, *F*(1,56) = .54, *p*<.47, *Partial Eta Squared* = .01, were significant.

**Table 3:** Means And Standard Deviations For Number Of Pens Picked Up.

	Super flight	Helicopter flight
**Helping**	13.69 (1.54)	12.26 (4.24)
**Touring**	13.43 (1.58)	10.56 (6.13)

Standard deviations are in parentheses.

### SSQ

We performed an ANOVA with task type and motion type as independent variables. There were no significant effects for simulator sickness (see Text D in File S1). Means and SDs are listed in Table A in File S1.

### Presence

Table 4 contains the mean and standard deviation of self-reported presence by condition. We ran an ANOVA with task type and motion type as independent variables. There was a marginally significant effect of motion type, F(1,56) = 3.74, p<.058, Partial Eta Squared = .06, such that flying participants reported more presence than helicopter participants. Neither the main effect of task type, F(1,56) = .33, p<.57, Partial Eta Squared = .01, nor the interaction, F(1,56) = 2.03, p<.16, Partial Eta Squared = .04, were significant.

**Table 4:** Means and Stanford Deviations for the Measure of Presence.

	Super flight	Helicopter flight
**Helping**	3.18 (.86)	3.27 (.62)
**Touring**	3.01 (.72)	3.65 (.68)

Standard deviations are in parentheses.

### Intention to Help

We performed an ANOVA with task type and motion type as independent variables. There were no significant effects (see Text D in File S1). Means and SDs are listed in Table B in File S1.

## Discussion

To sum up the results, flying participants were quicker to help than helicopter participants. In addition, there was a significant effect of number of pens picked up such that flyers picked up more pens than helicopter riders. In fact, six participants did not help at all, and these participants were all in the helicopter condition. The virtual power of flight facilitated subsequent helping behavior in the real world. However, there was somewhat of a ceiling effect in that the majority of participants, regardless of condition, helped.

Whereas much research has been done on whether and how violent videogames can lead to aggressive behavior (see Anderson [21] and Anderson, Shibuya, and colleagues [22]), this is the first study to document that the “next” technology in video gaming–virtual reality–has the potential to facilitate prosocial behavior by allowing players to become superheroes.

One hypothesized explanation for these results is that embodying the ability to fly in VR primes concepts and stereotypes related to superheroes in general or to Superman in particular, and thus facilitates subsequent helping behavior in the real world [23], [24]. Similarly, it is possible that embodying this power may do more than prime such concepts; it may shift participants’ self-concept or identity in a powerful way as “someone who helps,” at least briefly. Research supporting this hypothesis can be found in work on the role of self-concept in mediating the effect of concept activation on behavior [25], [26], [27]. (When the research results were shared with Paul Levitz, former DC Comics Publisher and President, and comic book editor and writer, Mr. Levitz noted that people familiar with superhero tropes implicitly know that after a character discovers a newfound superpower, the character’s task is to decide how to use it–for personal gain or for the greater good. Perhaps that implicit knowledge was operating in the current study, leading super flight participants to decide unconsciously and perhaps automatically to use their power for good.).

Our study’s results are particularly intriguing in that the experimenter and the materials in the current study did not use the word “superhero” or the prefix “super-” before the virtual experience, and the word “superhero” or prefix “super-” were never uttered during or after the experiment had ended. However, because the participants in the flying conditions were given a superhuman ability, cognitive channels linking “super” activity (and related concepts and stereotypes) to heroism and helping behavior may have been opened, which would then influence their decision to help. Future studies can elucidate the underlying mechanism(s), such as teasing out the extent to which the priming of “superhero” (or Superman specifically) leads to subsequent helping behavior, and the extent to which such priming may activate a change in self-concept and, through this change, subsequent helping behavior.

There was no main effect of task. One explanation for this lack of significance is that, despite the rich backstory for saving the child given to participants in both helping conditions, the actual saving task may not have been a vivid and immersive enough experience. Whereas participants were told they had a vial of insulin in their pocket, they did not see it or get to experience actually handing it to the child. Additionally, some participants noted that they felt that they didn’t truly “find” the child in the city. So spotting the child may have felt like happenstance rather than a saving moment. (Note that in this study, we did not perform a manipulation check to determine whether the instructions for the helping conditions were perceived by participants as we intended.).

Another psychological basis for the difference between the super flight and helicopter conditions might be explained by an *involvement* versus *observation* discrepancy. Said another way, participants in the super flight conditions were *active* agents in the VR world, and participants in the helicopter conditions were comparatively *passive* as passengers. A similar discrepancy was found in a study by Calvert and Tan [28]: Young adults who played an aggressive virtual reality game showed increased physiological arousal and aggressive thoughts compared to those who observed someone else playing the game. While all participants in our study were actively immersed in virtual reality, there can be parallels drawn between Calvert and Tan’s observation condition and our helicopter condition. One participant in a helicopter condition was noted as saying “I felt like the helicopter pilot really did all the work. I don’t think I helped.” From the perspective of participants in the helicopter condition, it may have seemed that they were watching someone else actively explore/navigate the virtual city (and, with the use of yoking, they were) and thus, they were merely observers in the virtual world. A future study should allow participants in the helicopter condition to actively control their navigation, thus bringing them out of the observer role, to see if similar results ensue.

The self-reported measure of orientation toward helping (the adapted POQ subscale) was not significant across groups. This lack of significance could be due to the hypothetical nature of many of the questions (e.g., “If a stranger left something behind, I would not tell him or her”) and the relatively infrequent opportunity to help in some of the specific ways described may have led participants to have difficulty accurately endorsing the frequency with which they would help in each of the specific ways. The lack of significance of this measure could also reflect the limitations of self-report measures in general. Bailenson, Aharoni, and colleagues [19] discuss the limitations of self-report and find, after a series of experiments, that results indicated by behavioral measures can be missed by self-report measures. The authors state that: “one of the greatest limitations in questionnaire-based studies is that participants are not always the most accurate judges of their own thoughts and feelings, so they often misreport affective and cognitive responses to stimuli. Therefore, dependent measures based on self-report questionnaires are best used in conjunction with other measures.” ([19] p. 7). Slater [20] reports a similar finding, pointing out that self-report measures in virtual reality often do not measure the construct they are designed to measure.

The results may also have been influenced by other factors, such as the effect of presence in VR: Participants in the flying condition had significantly higher scores on the measure of presence, indicating that they felt more immersed in the experience and that it felt more “real.” Previous research suggests that stronger levels of presence in VR are more likely to affect behavior in the real world [29]. While the higher levels of presence in the flying condition compared to the helicopter condition offers some support for the idea that presence may be contributing to the difference in helping, a mediation analysis of presence on helping resulted in no significant effects for either the time taken to help (correlation: *r* = .15) or the number of pens picked up (*r* = -.13). Another factor that may have influenced the results is the homogeneity of our participant population: college students in their late teens to early twenties.

Perhaps a different self-report measure and/or more behavioral measures of helping would elucidate the relationship between super flight and subsequent prosocial behavior. For instance, participants could be asked whether they’d like to volunteer to remain in the lab to help the researcher with a few more studies or if they would like to donate to a charity sponsored by the lab. Also, in this study we did not ensure that the experimenter was blind to condition. Future work should do so in order to prevent possible demand characteristics.

This study is one of the first to examine the effects of prosocial behavior in VR and the prosocial effects of embodying a superpower. Future studies might address the questions: Will allowing people to experience super flight for longer periods of time lead them to be more prosocial afterward? Is the prosocial effect limited to the virtual experience of *flight*, or might it arise after other “superpowers” as well? What about embodying a specifically identified superhero such as Superman? Finally, if our results are replicated, future studies can examine the specific mechanisms at work.

## Supporting Information

File S1 This file contains the following supporting information. **Text A** Full Experiment Script. **Text B** Environmental Presence Scale. **Text** **C** Adapted Subscale of Prosocial Orientation Questionnaire. **Text** **D** Inferential Statistics for Non-Significant Effects. **Table** **A** Means and Standard Deviations for Measure of Motion Sickness (SSQ). Standard deviations are in parentheses. Higher numbers indicate more sickness. **Table** **B** Means and Standard Deviations for Measure of Intention to Help. Standard deviations are in parentheses. Lower numbers indicate more helpfulness.
(DOC)
