# Supplementary material for: Virtual Superheroes: Using Superpowers in Virtual Reality to Encourage Prosocial Behavior
Source: PLoS One. 2013 Jan 30;8(1):e55003. doi: 10.1371/journal.pone.0055003 (PMC3559425; doi:10.1371/journal.pone.0055003)
Supplement: File S1 — This file contains the following supporting information. Text A Full Experiment Script. Text B Environmental Presence Scale. Text C Adapted Subscale of Prosocial Orientation Questionnaire. Text D Inferential Statistics for Non-Significant Effects. Table A Means and Standard Deviations for Measure of Motion Sickness (SSQ). Standard deviations are in parentheses. Higher numbers indicate more sickness. Table B Means and Standard Deviations for Measure of Intention to Help. Standard deviations are in parentheses. Lower numbers indicate more helpfulness. (DOC) [file pone.0055003.s001.doc]

**Supporting Information**

**Text A**

**Full Text of Experimental Instructions**

*This research study is investigating the effects of different kinds of exploration in immersive virtual reality environments. You will randomly be assigned to 1 of 4 conditions and will be given instructions as to your task for each condition.*

Flying Instructions (Conditions 1 and 3)

*In this virtual environment, you will have the ability to fly in a virtual city. You will have sensors placed on both of your hands. When you raise your arms, you will take off from the ground and fly higher in the air; and when you lower them you will float toward the ground. You will be able to direct your body by pointing your arms in the direction that you’d like to fly. In order to control speed, you will need to control the distance between your hands. The closer your hands get to each other, the faster you will fly; and conversely, the further apart your hands are, the slower you will fly. Let me know if you begin to feel uncomfortable or if you experience any motion sickness.*

Helicopter Instructions (Conditions 2 and 4)

*In this virtual environment, you will be a passenger in a helicopter, exploring a virtual city. You won’t be able to control the direction of the helicopter but you will be able to maneuver your head to look out the helicopter window. You will not be able to see the pilot, as he or she is seated behind the partition that separates the pilot and passenger seats. Let me know if you begin to feel uncomfortable or if you experience any motion sickness.*

Pre-Recorded Task Instructions, Condition 1: Super Hero with flying

*There has been an earthquake warning and the city has been evacuated. A child has been unaccounted for and the parents have informed authorities that their child is diabetic and will go into shock without insulin. You have a vile of insulin in your pocket. Your task is to fly through the city to find the child and deliver the insulin, saving the child’s life. As soon as you see the child, call out. You must indicate to the experimenter that you’ve found the child so please clearly announce that you see the child when the child comes into view. You may now begin your search. Lift your arms above your head to take off from the ground.*

Pre-Recorded Task instructions, Condition 2: Super Hero in helicopter

*There has been an earthquake warning and the city has been evacuated. A child has been unaccounted for and the parents have informed authorities that their child is diabetic and will go into shock without insulin. There is a vile of insulin in the helicopter. Your task is to use your vantage point to find the child in the city below and deliver the insulin, saving the child’s life. As soon as you see the child, call out. You must indicate to the experimenter that you’ve found the child so please clearly announce that you see the child when the child comes into view. Your search begins now.*

Pre-Recorded Task Instructions, Condition 3: Helicopter Tour

*In this environment, you will use your ability to fly to explore a virtual city. You may begin by lifting your arms above your head to take off from the ground.*

Pre-Recorded Task Instructions, Condition 4: Flying Tour

*In this environment, you will be a passenger in a helicopter as it explores the virtual city. Your helicopter tour will begin now.*

Child Ending

*Thankfully, you’ve reached the child before diabetic shock set in. You hand the syringe to the child, who injects the insulin. You’ve now completed your task and saved the child’s life.*

Tour Ending

*Thank you for exploring the virtual city. You have now completed your virtual navigation. Please have a seat while I put the equipment away.*

**Text B**

**Environmental Presence Scale**

These questions concern your experience in the virtual world. Please read each question carefully and choose the answer that best reflects your feelings.

**To what extent did you feel like you were really inside the city?**

- I felt extremely like I was really inside the city.
- I felt a lot like I was really inside the city.
- I felt moderately like I was really inside the city.
- I felt slightly like I was really inside the city.
- I did not feel like I was really inside the city at all.

**To what extent did you feel surrounded by the city?**

- I felt extremely surrounded by the city.
- I felt very surrounded by the city.
- I felt moderately surrounded by the city.
- I felt slightly surrounded by the city.
- I did not feel surrounded by the city at all.

**To what extent did you feel you really visited the city?**

- I felt extremely like I really visited the city.
- I felt a lot like I really visited the city.
- I felt moderately like I really visited the city.
- I felt slightly like I really visited the city.
- I did not feel like I really visited the city at all.

**To what extent did you feel that the city seemed like the real world?**

- I felt extremely that the city seemed like the real world.
- I felt a lot that the city seemed like the real world.
- I felt moderately that the city seemed like the real world.
- I felt slightly that the city seemed like the real world.
- I did not feel that the city seemed like the real world at all.

**To what extent did you feel like you could reach out and touch the objects in the city?**

- I felt extremely like I could reach out and touch the objects in the city.
- I felt a lot like I could reach out and touch the objects in the city.
- I felt moderately like I could reach out and touch the objects in the city.
- I felt slightly like I could reach out and touch the objects in the city.
- I did not feel like I could reach out and touch the objects in the city at all.

**Text C**

17-item adapted subscale of the Prosocial Orientation Questionnaire

Please choose the version of the statement that best describes you.

**I would welcome other students to join me when I study.**

- I would welcome other students to join me when I study all the time.
- I would welcome other students to join me when I study most of the time.
- I would welcome other students to join me when I study sometimes.
- I would rarely welcome other students to join me when I study.
- I would never welcome other students to join me when I study

**I would spend time and money to help those in need.**

- I would spend time and money to help those in need all the time.
- I would spend time and money to help those in need most of the time.
- I would spend time and money to help those in need sometimes.
- I would rarely spend time and money to help those in need.
- I would never spend time and money to help those in need

**I would try to stop my friends from arguing or fighting.**

- I would always try to stop my friends from arguing or fighting.
- I would try to stop my friends from arguing or fighting most of the time.
- I would try to stop my friends from arguing or fighting sometimes.
- I would rarely try to stop my friends from arguing or fighting.
- I would never try to stop my friends from arguing or fighting.

**I would volunteer to help charity if they need my help.**

- I would always volunteer to help charity if they need my help.
- I would volunteer to help charity if they need my help most of the time.
- I would volunteer sometimes to help charity if they need my help.
- I would rarely volunteer to help charity if they need my help.
- I would never volunteer to help charity if they need my help.

**I would help my family if they were in need.**

- I would always help my family if they were in need.
- I would help my family if they were in need most of the time.
- I would help my family if they were in need sometimes.
- I would rarely help my family if they were in need.
- I would never help my family if they were in need.

**If a stranger left something behind, I would tell him or her.**

- If a stranger left something behind, I would always tell him or her.
- If a stranger left something behind, I would tell him or her most of the time.
- If a stranger left something behind, I would tell him or her sometimes.
- If a stranger left something behind, I would rarely tell him or her.
- If a stranger left something behind, I would never tell him or her.

**I feel happy when I share my things with others.**

- I always feel happy when I share my things with others.
- I feel happy when I share my things with others most of the time.
- I sometimes feel happy when I share my things with others.
- I rarely feel happy when I share my things with others.
- I never feel happy when I share my things with others.

**I would offer to help people who are disabled (e.g. in a wheel chair).**

- I would offer to help people who are disabled all the time.
- I would offer to help people who are disabled most of the time.
- I would offer to help people who are disabled sometimes.
- I would rarely offer to help people who are disabled.
- I would never offer to help people who are disabled.

**I feel jealous when my friends do exceptionally well.**

- I never feel jealous when my friends do exceptionally well.
- I rarely feel jealous when my friends do exceptionally well.
- I sometimes feel jealous when my friends do exceptionally well.
- Most of the time, I feel jealous when my friends do exceptionally well.
- I always feel jealous when my friends do exceptionally well.

**I look down on my classmates who do not do well in school.**

- I never look down on my classmates who do not do well in school.
- I rarely look down on my classmates who do not do well in school.
- I sometimes look down on my classmates who do not do well in school.
- Most times, I look down on my classmates who do not do well in school.
- I always look down on my classmates who do not do well in school.

**I do things to make my parents happy.**

- I always do things to make my parents happy.
- Most times, I do things to make my parents happy.
- I sometimes do things to make my parents happy.
- I rarely do things to make my parents happy.
- I never do things to make my parents happy.

**I like to help my friends (for example, to return a library book for a friend).**

- I like to help my friends all the time.
- I like to help my friends most of the time.
- I like to help my friends sometimes.
- I rarely like to help my friends.
- I never like to help my friends.

**I would help my friends when they have a problem.**

- I would always help my friends when they have a problem.
- I would help my friends when they have a problem most of the time.
- I would sometimes help my friends when they have a problem.
- I would rarely help my friends when they have a problem.
- I would never help my friends when they have a problem.

**I would give up something I like to help my friends or family.**

- I would always give up something I like to help my friends or family.
- Most times, I would give up something I like to help my friends or family.
- Sometimes I would give up something I like to help my friends or family.
- I would rarely give up something I like to help my friends or family.
- I would never give up something I like to help my friends or family.

**I would give up my seat on a train or bus to someone in need.**

- I would always give up my seat on a train or bus to someone in need.
- I would give up my seat on a train or bus to someone in need most of the time.
- Sometimes, I would give up my seat on a train or bus to someone in need.
- I would rarely give up my seat on a train or bus to someone in need.
- I would never give up my seat on a train or bus to someone in need.

**I would help to do housework at home.**

- I would always help to do housework at home.
- Most of the time, I would help to do housework at home.
- I would sometimes help to do housework at home.
- I would rarely help to do housework at home.
- I would never help to do housework at home.

**I would not help someone who has not helped me.**

- I would not help someone who has not helped me every time.
- I would not help someone who has not helped me most times.
- I would not help someone who has not helped me sometimes.
- I would rarely not help someone who has not helped me.
- I would help someone who has not helped me.

**Text D**

**Inferential Statistics for Non-Significant Effects**

We ran an ANOVA with task type and motion type as independent variables and SSQ score as the dependent variable. There was no significant effect of motion type, *F*(1, 56) = .19, *p*<.66, *Partial Eta Squared* = .003. There was no significant effect of task type, *F*(1,56) = .16, *p*<.69, *Partial Eta Squared* = .003. There was no significant interaction, *F*(1,56) = 1.24, *p* <.27, *Partial Eta Squared* = .02.

We ran an ANOVA with task type and motion type as independent variables and intention to help score as the dependent variable. There was no significant effect of motion type, *F*(1, 56) = 1.93, *p*<.17, *Partial Eta Squared* = .03. There was no significant effect of task type, *F*(1,56) = .12, *p*<.73, *Partial Eta Squared* = .002. There was no significant interaction, *F*(1,56) = .03, *p* <.86, *Partial Eta Squared* = .001.

**Table A**

**Means and Standard Deviations for Measure of Motion Sickness (SSQ)**

Table S2. Means and Standard Deviations for Measure of Motion Sickness

|  | Super flight | Helicopter flight |
| --- | --- | --- |
| Helping | 1.39 (.24) | 1.26 (.35) |
| Touring | 1.33 (.32) | 1.38 (.35) |

**Table B**

**Means and Standard Deviations for Measure of Intention to Help**

Table S1. Means and Standard Deviations for the Measure of Intention to Help

|  | Super flight | Helicopter flight |
| --- | --- | --- |
| Helping | 1.87 (.35) | 2.00 (.26) |
| Touring | 1.91 (.34) | 2.01 (.32) |
